# Highly efficient multicolor multifocus microscopy by optimal design of diffraction binary gratings

**DOI:** 10.1038/s41598-017-05531-6

**Published:** 2017-07-13

**Authors:** Bassam Hajj, Laura Oudjedi, Jean-Bernard Fiche, Maxime Dahan, Marcelo Nollmann

**Affiliations:** 1 Laboratoire Physico-Chimie, Institut Curie, PSL Research University, CNRS UMR168, 75005 Paris, France; 2 Sorbonne Universités, UPMC Univ Paris 06, 75005 Paris, France; 3 Centre de Biochimie Structurale, CNRS UMR5048, INSERM U1054, Université de Montpellier, 29 Rue de Navacelles, 34090 Montpellier, France

## Abstract

Multifocus microscopy (MFM) allows sensitive and fast three-dimensional imaging. It relies on the efficient design of diffraction phase gratings yielding homogeneous intensities in desired diffraction orders. Such performances are however guaranteed only for a specific wavelength. Here, we discuss a novel approach for designing binary phase gratings with dual color properties and improved diffraction efficiency for MFM. We simulate binary diffraction gratings with tunable phase shifts to explore its best diffraction performances. We report the design and fabrication of a binary array generator of 3 × 3 equal-intensity diffraction orders with 74% efficiency, 95% uniformity and dual color capability. The multicolor properties of this new design are highlighted by two-color MFM imaging. Finally, we discuss the basics of extending this approach to a variety of diffraction pattern designs.

## Introduction

Fluorescence microscopy is a powerful tool to decipher biological processes in living cells. A complete understanding of proteins or organelles behavior requires a recording of their spatio-temporal behavior in their 3D cellular habitat. One approach for fast 3D imaging, called multiplane microscopy, consists in splitting the emission of an optical microscope into different axial images that are simultaneously recorded. Several multiplane microscopy modalities have been developed based on this principle. One such modality divides the light through beam splitters, and projects different axial images on several cameras^1, 2^. Other methods rely on opacity-based diffraction gratings or a spatial light modulator to divide the image into different diffraction orders while adding order-dependent defocusing^3–6^. The former approach requires a costly optical scheme as it requires multiple cameras, while the latter has a limited application field due to a reduced photon collection efficiency and chromatic aberrations. Recently, a new multiplane microscopy modality, called multifocus microscopy (MFM), has been developed. MFM enables fast and ultrasensitive 3D imaging^7^ and relies on the parallel acquisition of images of different focal planes on the same camera sensor without mechanical scanning. The MFM has proven to be beneficial for photon-demanding applications such as single molecule imaging and localization in the 3D environment of cells^8–12^. Fast and sensitive tracking of single emitters was achieved with an acquisition speed of up to 100 volumes per second over 4 microns of axial depth. This imaging depth matches the dimensions of cell organelles and is highly beneficial for volumetric super-resolution imaging using photo-activated localization microscopy (PALM) or stochastic optical reconstruction microscopy (STORM).

The MFM is based on the introduction of multiple diffraction optical elements and prisms on the emission path of a wide-field microscope. As the main element of MFM, a diffraction grating (Multi-Focus Grating or MFG) has the key role of splitting the light into nine diffraction orders with equal intensities but with different degrees of defocusing such that, once refocused with a lens on a camera, the diffraction orders correspond to nine, equally spaced, focal planes. For demanding applications such as single molecule imaging and super-resolution microscopy, optimizing the photon budget is crucial. This requires the use of diffraction gratings with high transmission efficiency, defined as the ratio of the sum of intensities in the desired diffraction orders over the whole incoming light intensity. In order to preserve the photon budget, phase gratings have been privileged. A second fundamental factor in designing MFGs is the ability of obtaining homogeneous intensity distributions in the different diffraction orders. This is measured by the ratio between the dimmer and the brightest diffraction orders (hereafter called uniformity). Optimal MFG designs require simultaneous optimization of these two parameters: efficiency and uniformity.

Such diffraction gratings also known as “Dammann gratings” split incoming waves into regular arrays of similar waves of equal intensity^13^. Different efficient grating designs and simulation algorithms have been discussed in the last two decades^14–17^. Most of these designs use etching depths with discrete phase steps of 2*πs*/*M* with *s* = 1, 2, 3... *M*, and *M* being the number of phase steps to be imprinted on the final physical grating. To date, the commonly produced MFGs have used two-phase steps (0 − *π*). Binary MFG designs with 3 × 3 diffraction orders reach diffraction efficiency of 67%, very close to the theoretical limit^7^. It was predicted that this limit may be surpassed by using binary gratings with other phase steps, but this prediction has never been tested or implemented^18^. A second version of the 3 × 3 grating used 8 phases with a theoretical efficiency of 89%. However, in practice, producing a multiphase grating that reaches such performance is extremely challenging and the reported efficiency was considerably lower (<80%)^19^. This is mainly due to limitations in the available technologies and the difficulties in the alignment of the three photolithography masks during the production process^20^. Moreover and due to the wavelength dependent diffraction efficiency, (0 − *π*) binary or multiphase gratings are suitable only for a specific wavelength.

Here we show that highly efficient binary phase gratings can be designed and tuned to perform at two specific wavelengths. To this end, we simulated diffraction binary gratings in which the phase levels are 0 and *ϕ*, with *ϕ* not necessarily equal to *π*. Over the range of useful phase shifts, a diffraction efficiency of 74% was obtained for 3 × 3 diffraction orders, an intermediate performance between the previously reported ones for binary and multiphase gratings. Moreover, we show that 0 − *ϕ* gratings exhibit interesting tunable spectral properties with potential applications for dual color imaging. Finally, we expand our findings to different diffraction orders distributions.

## Methods and Results

### Simulations

**Figure 1:**
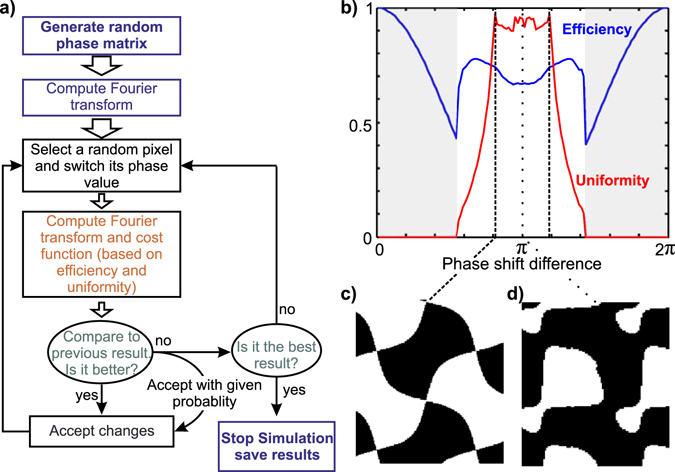
Simulation pipeline for phase grating. (**a**) Diagram for computational iterations to generate phase grating profiles for specific spot distribution. (**b**) Diffraction efficiency and uniformity of spots intensity for 3 × 3 array generator reported for each solution found for a specific phase shift. (**c**) The obtained phase grating motif, white and black colors correspond to 0 and 0.84 × *π* phase shifts simultaneously. (**d**) The simulated grating motif for a *π* phase shift.

A two-dimensional phase grating has the ability to diffract a beam of light into several diffraction orders. For MFM, an important task is to design a phase grating diffracting the beam into a MxN array of discrete diffraction orders with equal intensity centered at the 0^th^ order, and with the maximum attainable efficiency. Our simulation approximates the phase mask as a finite pixelated matrix. The value of each element in the matrix corresponds to the phase value of the pixel. Fourier transform was used to compute the diffraction pattern generated by the phase element. The grating shape design, i.e. the phase distribution, is expected to maximize the diffraction efficiency yielding the best photon collection (efficiency) while preserving equal intensity distribution between the orders of interest (uniformity). As such, a cost function is computed as $cost={\sum _{i,j}|{T}_{i,j}-{O}_{ij}|}^{7}$, based on the comparison of the desired target intensity distribution *T* to the obtained one *O*. (*i*, *j*) are the index of the matrix elements. The power of 7 guarantees a good convergence of the simulations towards the optimal grating design (See Supplementary Fig. S1). During simulation, we iterated the grating shape by random pixel switching between 0 and a fixed phase shift value $\varphi$ to reach the best diffraction performance. Furthermore, we used simulated annealing, a standard method to ensure convergence of the simulation algorithm towards a global minimum (as opposed to a local minimum). This implementation is based on accepting pixel flipping even if they yield increased cost values. These exceptions are more frequent at the beginning of the simulations and become scarcer with time as temperature is lowered. In our simulations, temperature was decreased linearly at each iteration. The exception probability decreases exponentially with simulation time, assuming that the main divergence in solutions is mostly obtained at the beginning of the simulation process. In practice exception was allowed if ${e}^{({{\rm{Cost}}}_{k-1}-{{\rm{Cost}}}_{k})/Temperature}$ was higher than a random number between 0 and 1. Here *k* is the iteration number. The iterations were repeated until no further improvement was obtained (Fig. 1(a)).

We first considered the problem of a 3 × 3 array generator. The cost function was computed over the 9 central orders. In each simulation, we fixed the value of the phase step *ϕ* and computed the best possible solution. All possible values of the phase steps between 0 and 2*π* were explored for a binary grating. In practice, this phase shift *ϕ* is translated into a physical etching depth *T* in the glass substrate:

1 $$T=\frac{\varphi \lambda }{2\pi (n(\lambda )-1)}$$

where *λ* is the wavelength and *n*(*λ*) the index of refraction of the substrate at this wavelength. The phase shift *ϕ* was varied in discrete steps of *π*/64, corresponding to the range of the physical etching errors (~10–20 nm) of current dry etching techniques. The best efficiency and uniformity values obtained by simulation were traced in Fig. 1(b) as a function of the phase shift. The efficiency and uniformity were symmetrical around the *π* phase shift. This can be expected from the Fourier transform symmetry for *ϕ* and 2*π* − *ϕ* phases (only the sign of the amplitude changes). This interesting property was never explored before, and proved to be very useful as explained later. We obtained a theoretical efficiency of 67% for a *π* phase shift binary grating (Fig. 1(c)), as previously shown^7^. However, the best performance exceeded 74% efficiency and 97% uniformity for a 0.84 × *π* phase shift (and for the symmetric value 1.18 × *π* within the error range) (Fig. 1(d)). Throughout this paper the gratings shown are formed by a 2 × 2 repetition of the main motif. The main difference between the *π* phase shift motif and the 0.84 × *π* design is the regularity of the shape. The latter is more symmetric and regular, but has sharper connection edges.

**Figure 2:**
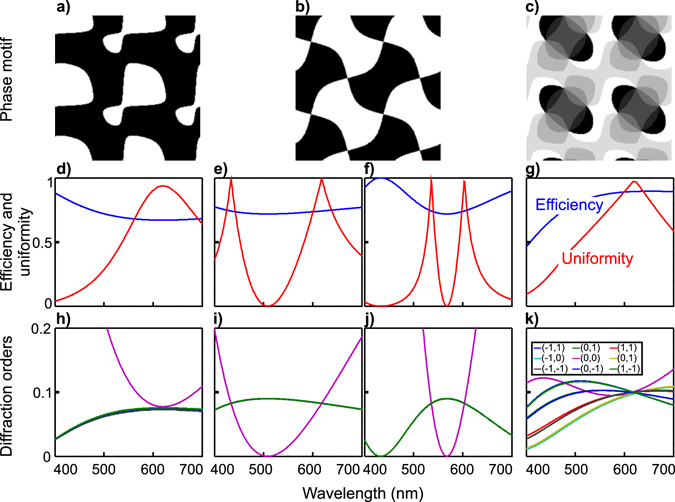
Influence of the wavelength on the diffraction performance. (**a**–**c**) Are the investigated 3 different gratings for generating 3 × 3 equal intensity diffraction orders array. (**a**) corresponds to binary phase shift of *π*, (**b**) 0.84 × *π* and (**c**) is an 8 phase grating. In (**d**–**g**) are presented the corresponding efficiency and uniformity as function of the wavelength while (**h–k**) details the different order intensities. The difference between (**e** and **i**) from one side and (**f**) and (**j**) on the other, is that the former are simulated for 0.84 × *π* phase shift and the latter are for 0.84 × *π* + 2*π*.

To further explore the impact of grating shape on diffraction performance, the efficiency and uniformity were computed as a function of the diffracted wavelength. The index of refraction dispersion as function of the wavelength was computed using Sellmeier’s equation for a fused silica substrate^21^. Figure 2(a) shows the *π* − binary grating motif, Fig. 2(d) the corresponding efficiency and uniformity, and Fig. 2(h) the intensities for the different diffraction orders as a function of the imaging wavelength. As this paper is oriented towards fluorescence imaging, we only show the visible wavelength range. In the simulation, we assume that the etching depth was chosen for an optimal performance around 620 nm (center wavelength for mCherry imaging, for instance). For this grating, the different diffraction orders followed the same wavelength behavior except the central one which is responsible for the change in uniformity. The efficiency was fairly constant over the visible range.

In contrast, for a 0.84 × *π* grating the uniformity changed more rapidly with wavelength (Fig. 2(b) and (e)). This is mainly due to the strong dependence of the central order intensity on the wavelength. The other diffraction orders varied more smoothly over the visible range (Fig. 2(i)). This yielded a wavelength bandwidth over which the grating can be used that was on the order of ~40 nm. The efficiency on the other hand is stable over the visible range. Interestingly, a second band at a different wavelength (centered around 435 nm) exhibited the same uniformity behavior. A closer investigation showed that the second wavelength band corresponds to the second peak of efficiency at 1.18 × *π* for which the same grating motif was obtained. In fact, the physical etching depth producing a 0.84 × *π* phase shift at 620 nm wavelength yields a phase shift of 1.18 × *π* at 435 nm wavelength. This means that due to this symmetry, and in contrast to the *π* − grating, the same 0.84 × *π* − grating can be used to image efficiently at two different wavelengths. This property is the second important feature of this grating, in addition to its increased transmission efficiency.

Moreover, due to the periodicity of the phase shift effect, we can expect other wavelength bands of similar efficiencies and uniformities at smaller wavelengths. Those wavelengths *λ*′ satisfy the following relationship:

2 $$\frac{\lambda ^{\prime} }{n(\lambda ^{\prime} )-1}=\frac{\lambda }{n(\lambda )-1}\times \frac{\varphi }{\varphi +2m\pi }$$

where *λ* and *n* (*λ*) correspond to the design wavelength and the corresponding index of refraction of the substrate, respectively. *ϕ* is the phase shift the grating was etched for, and *m* is an integer number *m* = 1, 2, 3... Due to nonlinear dispersion, the index of refraction increases faster for shorter wavelengths, predicting that the two peaks at 620 nm and 435 nm would become narrower and closer to each other for shorter wavelengths. Thus, it is possible to tune two wavelength bands for the same grating provided that the grating is etched for a longer wavelength with an etching thickness equivalent to a phase shift of 0.84 × *π* + 2*pπ*, *p* = 1, 2, 3... at the desired wavelength. For *p* = 1 the imaging bands become closer (centered at 620 nm and 530 nm), but the useful bandwidth is reduced (Fig. 2(f) and (j)).

Finally, we compared this grating to a multiphase grating design. Figure 2(c) shows the 8-phase motif that was previously reported^19^. For this multiphase motif, the wavelength influences all the diffraction orders, but each order is affected differently thus yielding a highly wavelength-sensitive grating (Fig. 2(g) and (k)). For multicolor imaging applications, multiple gratings are thus necessary, each etched to the correct phase shift.

To illustrate the advantage of improved efficiency and multicolor properties of our new grating design, a binary 0.84 × *π* grating was implemented for multifocus microscopy. In this context, the main motif had a physical dimension of 12 µm repeated over a large area (~5 mm diameter), matching the back pupil size of the objective. We designed a grating for a 100X, oil immersion, 1.4 NA objective, commonly used for single-molecule imaging. The design imaging wavelength was fixed to be centered at 620 nm. Following the sine condition, a distortion is applied on the motif in a space-varying controlled manner to induce an order-dependent defocusing leading to a Δ*z*_*d*_ = 450 *nm* spacing between imaging planes for an imaging wavelength centered at 620 nm. This distortion along the x direction follows the equation:

3 $$\delta x={n}_{\lambda }\frac{d}{\lambda }{\rm{\Delta }}{z}_{d}\sqrt{1-\frac{{x}^{2}+{y}^{2}}{{({n}_{\lambda }{f}_{obj})}^{2}}}$$

Where *n*_*λ*_ is the index of refraction at the design wavelength *λ*, *d* the period, *f*_*obj*_ the objective focal distance and (*x*, *y*) are the coordinates at the pupil plane. The distortion in the y direction is *δy* = 3 × *δx* as previously explained^7, 8^. This distortion results in a diffraction-order dependent spherical aberration correction. Other aberrations, such as inherent to the microscope or sample-induced are not accounted for during the simulation and design process. They equally influence the image quality in conventional microscopy and MFM. Such additive phase can be corrected with adaptive optics.

### Fabrication

The MFG was fabricated in a clean room environment using conventional photolithography and dry-etching techniques and following the procedure described elsewhere^12^.

**Figure 3:**
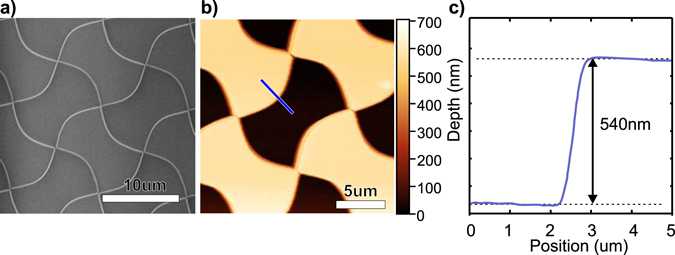
Fabrication and characterization of the 0.84 × *π* grating. (**a**) Electron microscopy image of the resulted grating showing a good border definition of the different etching zones. (**b**) Scanning AFM image of the heights of the gratings as a convolution of the physical size of the tip and the real structure of the grating. The cross section along the blue line is presented in (**c**).

The newly fabricated MFG was characterized by scanning electron microscopy (SEM) to ensure that the dimensions and aspect ratio of the grating pattern corresponded to the expected theoretical values (Fig. 3(a)). In addition, AFM microscopy was used to confirm SEM observations (Fig. 3(b)) and to estimate with nanometer precision the etch depth of the grating (Fig. 3(c)). In the present example, we measured a depth of 540 nm that corresponds to a phase shift of 0.84 × *π* at 620 nm wavelength. Supplementary Fig. S2 shows the influence of etching error on the performance of the MFG.

### Optical characterization

**Figure 4:**
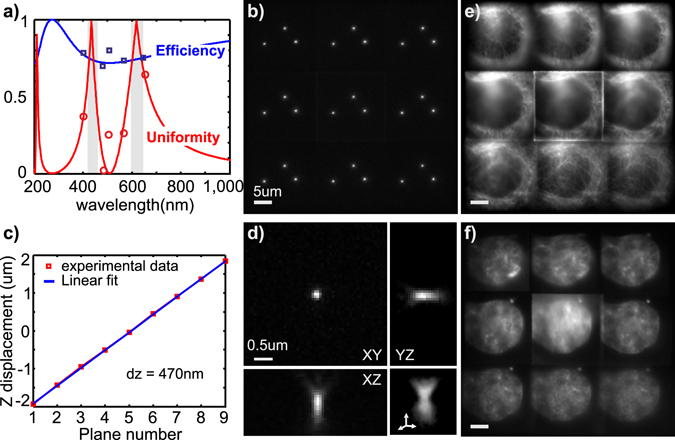
Optical characterization and validation of the fabricated 0.84 × *π* grating. (**a**) Simulations of the efficiency and uniformity of the diffraction through the grating as function of the wavelength. Overlaid are the measured values of the efficiency (blue squares) and uniformities (red circles). (**b**) Maximum intensity projection of a Z stack of fluorescent 200 nm diameter beads when imaged using multifocus microscope. (**c**) Validation of the plane spacing as measured by the beads. The spacing was found to be 470 nm for an emission centered at 620 nm in good agreement with the design value. (**d**) Zoom in on a point spread function of on the imaging planes, showing a diffraction limited PSF with no additional aberrations due to the grating motif. (**e**) Multifocus image of tubulin fibers in U2OS cells labeled with TMR molecules. (**f**) Multifocus image of the nuclear DNA labeled with Hoechst. The two images are taken with the same multifocus multicolor grating.

The fabricated optical element went through two steps of characterization. First, the intensities in the nine central diffraction orders were measured. The measurements were made with the laser lines 405, 488, 560 and 640 nm. The efficiency and uniformity are reported on Fig. 4(a) and overlaid on the theoretical prediction curve. Measurements agreed very well with the simulated data.

In a second step, the grating was placed in the multifocus microscope emission path, conjugated with the back focal plane of the microscope objective as previously described^7^. The grating splits the emission light into the 3 × 3 diffraction orders, which are tiled and imaged simultaneously on the camera. Furthermore, the grating is distorted resulting in additional, order-dependent, degree of defocusing. Distance between imaging planes was measured as follows: (1) Fluorescent beads were placed on a coverglass and imaged using a 620 nm emission bandpass filter; (2) The sample was moved axially in discrete steps over the imaging volume using a piezoelectric stage. A maximum intensity projection is shown in Fig. 4(b); (3) The axial separation between consecutive planes is computed by determining the axial position at which the beads intensity was maximal (see ref. 8 for the detail description) (Fig. 4(c)). From these measurements, we found a spacing of 470 nm when the imaging bandwidth is centered at 620 nm, which compares well with the design specifications (450 nm). We note that the spacing was defined for a 620 nm wavelength. Imaging at a different wavelength such as at 434 nm (the second uniformity peak), would result in a different observed spacing as it is expected by theory^8^. For dual color imaging, this can be accounted for in a post-processing step. Measurement of the point spread function in each plane rules out grating-induced additional aberrations (Fig. 4(d)).

Direct measurement of the transmission efficiency at the design wavelength of 620 nm was not possible due to the lack of laser lines at this wavelength. Thus, we inferred this value by estimating the uniformity when imaging fluorescent beads through a bandpass filter centered on 620 nm. Uniformity approached 86% as a mean value, yielding an estimated efficiency >73.5% over the imaging band. This efficiency was very close to the design specification of 74%, and higher than previous binary designs (67%). We note that in fluorescence imaging, a broad wavelength band is imaged in contrast to the narrow line of a laser. As shown in Supplementary Fig. S3, the wavelength dependency of the efficiency and uniformity is barely modified for broad wavelength bandwidths (50–100 nm), typical for fluorescence filters.

Finally, to illustrate the multicolor properties of this grating we performed two-color 3D wide-field imaging. U2OS cells were plated on a coverglass and transfected to express tubulin fused to Halo-tag. Cells were labeled with TMR Halo ligand before fixation in paraformaldehyde (PFA) at room temperature. As a marker for DNA, Hoescht was introduced in a second step. Two color volumetric images were acquired. Tubulin was imaged by exciting TMR at 560 nm and collecting the emission with a bandpass filter centered at 620 nm (Fig. 4(e)). DNA was sequentially imaged by exciting Hoescht with a 405 nm laser line and an emission bandpass filter centered at 490 nm (Fig. 4(f)). The same grating was used to image both colors. A dichroic mirror splitted the emission on two distinct optical paths. Two cameras acquired the images independently for each channel. The image quality was preserved for the two channels. We note that the uniformity in the Hoescht channel is reduced because the collection filter was not centered at the optimal emission wavelength.

### Expanding to other array generator designs

**Figure 5:**
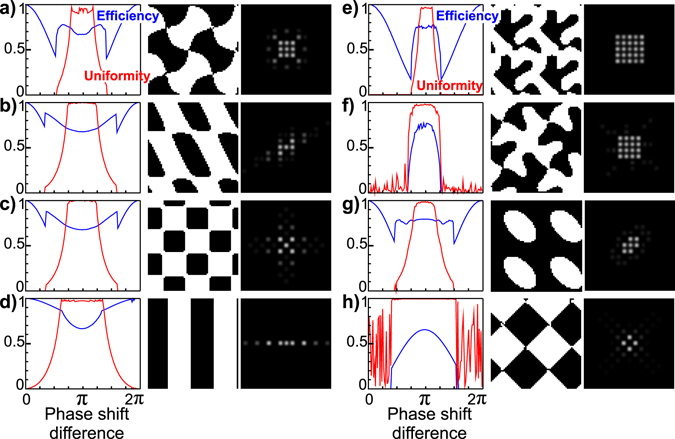
Simulation results for generating different diffraction orders distribution. From (**a**) to (**h**) are shown consecutively the optimization results for generating 9, 5, 5, 3, 25, 16, 7 and 4 equal intensity diffraction orders. For each case, the efficiency and uniformity are plotted against the phase shift difference. The best performing grating is presented, followed by the resulting diffraction orders distribution. The best designs were obtained for phase shifts different than *π* in (**a**–**d**) cases, while for (**e**–**h**) *π* phase shift resulted with the best performance.

In the previous example, we showed that by shifting the phase value from *π*, we could obtain better diffraction efficiency for a 3 × 3 array generator and gain dual color properties. Depending on the application type, the number of diffraction orders and thus imaging planes can be tuned. For instance, to image smaller volumes we can reduce the number of planes and thus increase the number of photons per plane. We therefore extended our investigation to cover a variety of diffraction orders distributions. The results are grouped in Fig. (5). The same simulation pipeline described beforehand was used to generate the different phase profiles. For each case, the *cost* function was adjusted according to the desired distribution of diffraction orders intensities. For example in the case of 5 × 5 diffraction orders intensity distribution, the *cost* function was evaluated over the zero order and +/− 1 and +/−2 diffraction order. For 4 × 4, the *cost* function was computed over the +/− 1 and +/−3 diffraction orders. We found three different behaviors in our simulations.

In the case of 9 (3 × 3), 5 and 3 diffraction orders intensity distribution, the efficiency can be enhanced for phase steps other than *π*. In the case of 25 (5 × 5), 16 (4 × 4) and 7 orders no improvement in efficiency could be obtained. However, for phase shift difference *ϕ* in vicinity of *π*, the efficiency and uniformity are preserved at their highest values. In a similar manner to the 0.84 × *π* grating, designing a grating at a phase shift of *π* ± Δ*ϕ* (where Δ*ϕ* does not exceed 0.1 × *π* for these cases), two wavelength-bands appear with similar maximum efficiency performances. Practically, this fact underlies that by changing Δ*ϕ* value, the two bands can be tuned to the desired imaging wavelengths.

**Figure 6:**
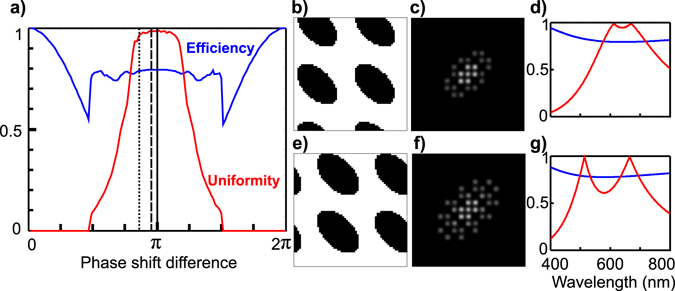
(**a**) Efficiency and uniformity of simulated diffraction gratings for 7 planes generation as a function of the binary step phase-shift. Vertical lines positions: plain: *π*, dashed: 0.95 × *π*, dotted: 0.86 × *π*. (**b**) The physical shape obtained for the 0.95 × *π* phase shift (**c**) the corresponding diffraction pattern, (**d**) the wavelength dependency of the efficiency and uniformity showing dual bands at 612 nm and 670 nm. (**e**) The physical shape obtained for the 0.86 × *π* phase shift, (**f**) the corresponding diffraction pattern, (**g**) the wavelength dependency of the efficiency and uniformity showing dual bands at 514 nm and 670 nm.

To illustrate this principle, we considered a 7-plane grating. Figure 6(a) shows the best obtained efficiency and uniformity as a function of the etched phase shift. For a phase shift of *π*±Δ*ϕ* (Δ*ϕ* < 0.2 × *π*), the two parameters are preserved. To illustrate the advantages of a grid design with a phase slightly shifted from *π*, we compared two grating motifs for 0.86 × *π* (Fig. 6(b) and (c)) and 0.95 × *π* (Fig. 6(e) and (f)) phase shifts. The contrast and brightness of the diffraction pattern in Fig. 6(c) and (f) was enhanced to properly visualize the difference in the diffraction behavior of the two designs. We note that these gratings (Fig. 6(b) and (e)) have a complementary phase profile (inversed phase profile) compared to the one in Fig. 5(g). Physically, these three motifs were very similar but exhibited slight roundness differences in their oval shapes in addition to the difference of phase shift (i.e. etching depth). Moreover, the complementarity does not alter the diffraction efficiency or uniformity in principle (see Supplementary Fig. S4). In fact two complementary designs with the exact motif shape can be obtained for the same imposed phase shift during simulation, and can be explained by a simple difference in the algorithm convergence procedure.

We plotted the values of the efficiency and the uniformity for the two designs obtained for 0.86 × *π* and 0.95 × *π* phase shifts, as a function of wavelength (Fig. 6(d) and (g)) assuming that gratings were designed for imaging at 670 nm. In both cases, a band of good performance was obtained at the design wavelength. In addition, for phase shifts different than *π*, a second wavelength band appeared with a similar performance but placed at a relative position that increased with phase shifts moving away from *π*. In the specific cases of 0.86 × *π* and 0.95 × *π* phase shifts, the two bands were centered at (514, 670 nm) and (612, 670 nm) respectively. Thus, it is possible to tune the two imaging wavelength bands when designing the grating without altering the efficiency and uniformity of the diffraction orders.

As a more general expression, the grating motif can be simulated for a phase shift of

4 $$\varphi =\frac{2\pi {\lambda }_{1}}{({\lambda }_{1}+{\lambda }_{2}\frac{n({\lambda }_{1})-1}{n({\lambda }_{2})-1})}$$

yielding a phase grating with equivalent diffraction properties at the two wavelengths *λ*_1_ and *λ*_2_. *n* is the index of refraction of the substrate.

Another interesting case is that of gratings with 4 diffraction orders. In this case, its wavelength performance is broad thus making it less sensitive to wavelength variation. We note here that in the case of 2 × 2 and 4 × 4 diffraction intensity distribution (or any even number of diffraction orders), the *π* phase shift grating results in the best diffraction performance. This can be explained by the need to diminish/eliminate the central order intensity. To this end, half of the beam going through the pattern into the zero order should be shifted by *π*. The two parts of the beam of equal area (shifted and non-shifted), will interfere destructively in the zero order. This is not a necessary condition in the case of an odd number of diffraction orders where the zero order should remain and phase shifts other than *π* can result in the overall diffraction efficiency improvement.

### Conclusion and perspective

In this paper, we investigated how the diffraction transmission efficiency and uniformity of binary MFGs depend on the design phase shift. Simulations with varying phase shifts were implemented and yielded an optimal performance for a step of 0.84 × *π* for a 3 × 3 array generator. This design predicted a diffraction efficiency of 74%, higher than previous binary designs. We validated this prediction experimentally by fabricating a MFG that was up to specifications and that delivered the expected transmission efficiency increase. Thus, this MFG design maintains the simplicity of microfabrication of previously reported binary designs while attaining a transmission efficiency comparable to that obtained in practice for multiphase MFG implementations (typically ~75% in our hands, unpublished).

Interestingly, the new grating design also displays dual color properties. In contrast to previously reported binary and multiphase designs, our MFGs exhibit two bands of equivalent performances in the visible range. The tradeoff is that the two best performing windows are narrower than with standard *π*-binary MFGs. These two bands can be tuned to the desired wavelength by etching to a depth equivalent to 0.84 × *π* + 2 *mπ*. It is also possible to introduce more bands into the visible spectrum. Such etching strategy can be applied, in principle, to previous implementations of binary and multiphase gratings to induce multicolor properties. However, in those cases, as the main two bands are far away from the secondary bands (further in the deep UV), the etching depth necessary to bring them to the visible range may become extremely large. Available lithography techniques might not be able to reach an acceptable surface quality and etching precisions at such depths.

Next, we demonstrated an implementation of the 0.84 × *π* grating in the context of multifocus microscopy for instantaneous volumetric imaging. The dual color performance was validated by imaging fluorescent beads and organic dyes in fixed cells. The uniformity of the different planes can be considered as a good proof of the grating dual band performance.

Varying the number of imaging planes with a different grating design can also benefit from phase shift variation. We have shown that diffraction efficiency and dual color properties can be gained by shifting the etching depth for gratings generating 9, 5 and 3 planes. For those examples, and for other cases where little difference was observed, designs optimized for *π* ± Δ*ϕ* phase shift could be used to tune the two bands of wavelengths with good diffraction performance. Depending on the application, if the uniformity is not crucial, other grating designs may be used with better overall efficiency.

We have shown here a general approach for efficient binary grating design and tuning. The dual color properties of the proposed grating would lower the technology cost and facilitate the accessibility of the technology to a wider range of users.

## Electronic supplementary material

Supplementary information
