# Supplementary material for: Highly efficient multicolor multifocus microscopy by optimal design of diffraction binary gratings
Source: Sci Rep. 2017 Jul 13;7:5284. doi: 10.1038/s41598-017-05531-6 (PMC5509674; doi:10.1038/s41598-017-05531-6)
Supplement: Supplementary file 1 — Supplementary information [file 41598_2017_5531_MOESM1_ESM.pdf]

Supplementary informations

Highly efficient multicolor multifocus microscopy by optimum design of diffraction binary gratings

Bassam Hajji[1,2,4], Laura Oudjedi[3], Jean-Bernard Fiche[3], Maxime Dahan[1,2,5], and Marcelo Nollmann[3,6]

[1] *Laboratoire Physico-Chimie, Institut Curie, PSL Research University, CNRS UMR168, 75005, Paris, France*

[2] *Sorbonne Universités, UPMC Univ Paris 06, 75005, Paris, France*

[3] *Centre de Biochimie Structurale, CNRS UMR5048, INSERM U1054, Université de Montpellier, 29 Rue de Navacelles, 34090 Montpellier, France*

[4] *bassam.hajj@curie.fr*

[5] *maxime.dahan@curie.fr*

[6] *marcelo.nollmann@cbs.cnrs.fr*

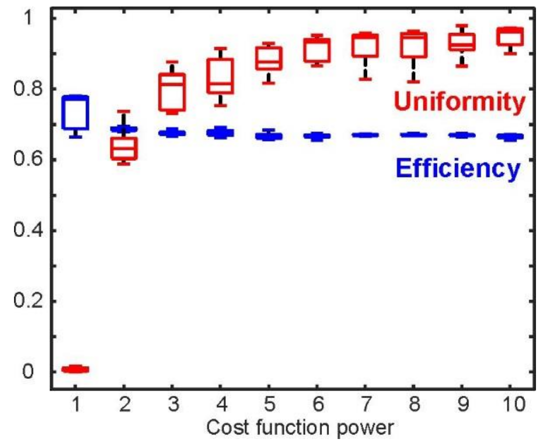

Figure S1: This figure shows the results of uniformity and efficiency obtained with different powers of the cost function. Simulations were performed for a 3x3 diffraction pattern grating with 32x32 pixels, and were repeated over 30 times yielding small differences in the uniformity values. The result is shown as boxplots. Simulations reached the best uniformities and efficiencies values for a cost function with power above 6. Higher exponents yielded equally good results. We made the choice to use power 7 throughout the different simulations.

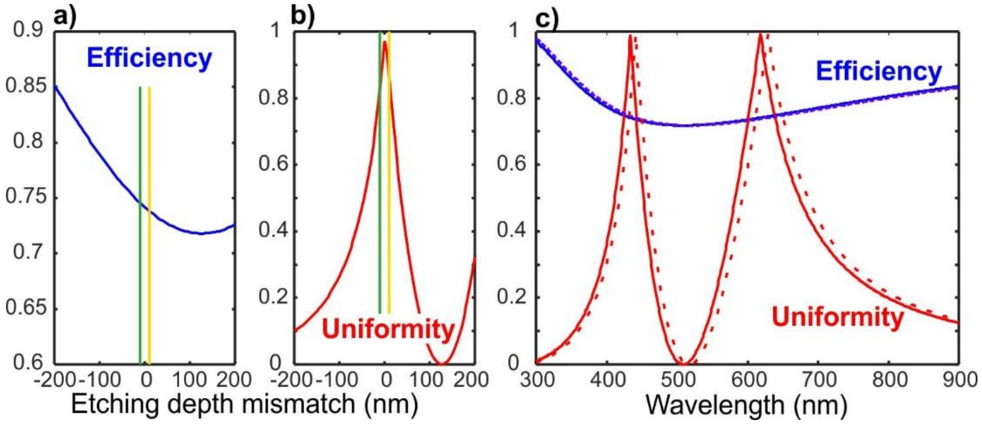

Figure S2: Figure a) and b) show the etching error influence on the efficiency and uniformity of the diffraction grating. The green and yellow lines correspond to and etching error of + and -10nm. Within this error the efficiency changes by ~0.5% and uniformity by less than15%. Thus, an error of +/-10nm is acceptable. Figure c) shows the efficiency and uniformity as a function of the imaging wavelength (solid lines) as compared to those obtained when a 10nm etching error is commit (dotted lines).

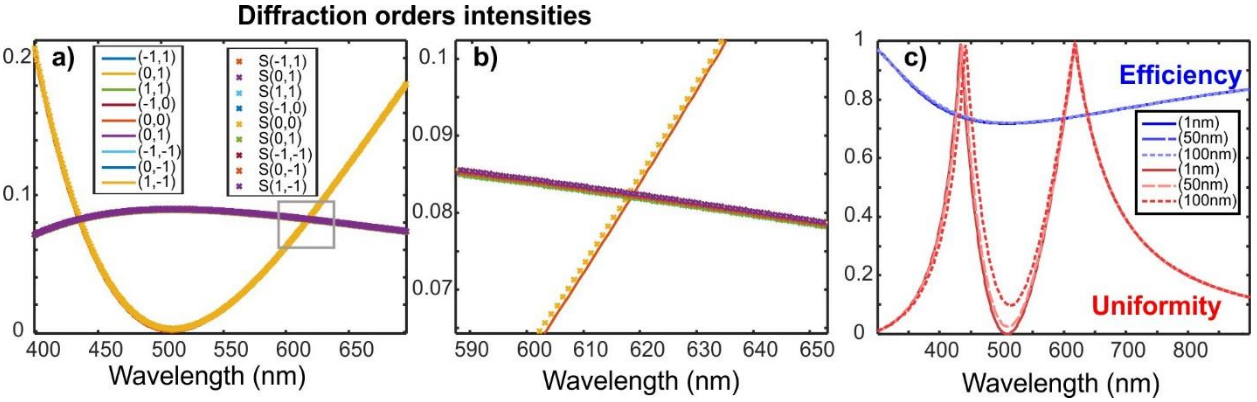

Figure S3: The figure a) shows the different diffraction orders intensities as a function of the wavelength. The lines are obtained without any integration, the crosses are obtained by integration over a 50nm bandwidth. b) is a zoom on the gray box in a), we can see that the integration do not alter that much the orders intensities as it can be considered linear in small region. In c) we show the computed efficiency and uniformity obtained with no integration (solid lines), with 50nm bandwidth integration (dashed lines) and with 100nm bandwidth integration (doted lines). It is clear that the influence of integration is very small with 50nm bandwidth (comparable with the emission filters characteristics that we use). The main difference is around 510nm, where the uniformity becomes higher due to the integration with larger band of wavelengths. Another difference was observed with a slight shift of the uniformity peak at 434nm to 435nm which is very negligible. We note however that in fluorescence imaging, the emission of the molecule is not constant across the imaging bandwidth. This explain the small reduction in uniformity that we observe when imaging fluorescence beads at 620nm (86%uniformity, instead of 98% theoretically) using the MFM.

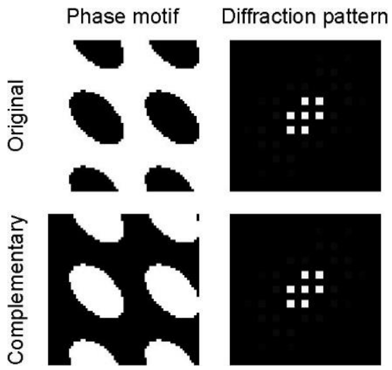

Figure S4: The diffraction pattern is the same for a phase motif and its complementary phase. The same order intensities are obtained in both cases.
